# Differential discounting in the economic evaluation of healthcare programs

**DOI:** 10.1186/s12962-019-0196-1

**Published:** 2019-12-17

**Authors:** Jürgen John, Florian Koerber, Mareike Schad

**Affiliations:** 1 Institute for Health Economics and Health Care Management, Helmholtz Zentrum München, German Research Center for Environmental Health (GmbH), Neuherberg, Germany; 2 GSK, Prinzregentenplatz 9, 81675 Munich, Germany; 3 Independent Researcher, Grüner Weg 2, 88339 Bad Waldsee, Germany

**Keywords:** Economic evaluation, Healthcare, Social discount rate, Differential discounting, Ramsey’s optimal growth model

## Abstract

**Background:**

The question of appropriate discount rates in health economic evaluations has been a point of continuous scientific debate. Today, it is widely accepted that, under certain conditions regarding the social objective of the healthcare decision maker and the fixity of the budget for healthcare, a lower discount rate for health gains than for costs is justified if the consumption value of health is increasing over time. To date, however, there is neither empirical evidence nor a strong theoretical a priori supporting this assumption. Given this lack of evidence, we offer an additional approach to check the appropriateness of differential discounting.

**Methods:**

Our approach is based on a two-goods extension of Ramsey’s optimal growth model which allows accounting for changing relative values of goods explicitly. Assuming a constant elasticity of substitution (CES) utility function, the growth rate of the consumption value of health depends on three variables: the growth rate of consumption, the growth rate of health, and the income elasticity of the willingness to pay for health. Based on a review of the empirical literature on the monetary value of health, we apply the approach to obtain an empirical value of the growth rate of the consumption value of health in Germany.

**Results:**

The empirical literature suggests that the income elasticity of the willingness to pay for health is probably not larger but rather smaller than 1 and probably not smaller but rather larger than 0.2. Combining this finding with reasonable values of the annual growth rates in consumption (1.5–1.6%) and health (0.1%) suggests, for Germany, an annual growth rate of the consumption value of health between 0.3 and 1.5%.

**Conclusion:**

In the light of a two-goods extension of Ramsey’s optimal growth model, the available empirical evidence makes the case for a growing consumption value of health. Therefore, the current German practice of applying the same discount rate to costs and health gains introduces a systematic bias against healthcare technologies with upfront costs and long-term health effects. Differential discounting with a lower rate for health effects appears to be a more appropriate discounting model.

## Background

Throughout the last few decades, there has been much debate on discounting of costs and health effects in the economic evaluation of healthcare interventions [1–8]. This debate was particularly controversial in the context of preventive measures, such as vaccination, due to their intertemporal character. Since costs usually occur upfront, whereas cost savings and health gains often manifest many years later, the choice of the discounting approach may exert a strong influence on the evaluation results [9–11]. Additionally, there are various normative arguments, such as intergenerational justice, which complicate the matter even more [5, 10]. Dominant questions in this debate include the choice of a particular discounting model, such as constant discounting or a time-declining discount rate schedule, and whether or not to apply the same discount rates to future costs and health effects.

In this paper, we focus exclusively on differential discounting. The article is structured as follows: We start with discussing the pros and cons of differential discounting. We present the widely accepted proposition that a lower discount rate for health gains than for costs is justified if the consumption value of health (v_H_) is growing over time. In addition, we briefly review the arguments provided in support of an increase of v_H_. In “Methods” section, we present the two-goods extension of Ramsey’s optimal growth model as an approach to account for an increasing v_H_. The “Results” section contains the findings of our attempt to make use of this model in order to gain an empirical estimate of the growth rate of v_H_ in Germany. The last two sections of the article provide a discussion of our findings and a short conclusion.

### Arguments for and against differential discounting

To date, equal discounting of costs and health gains has been the dominant practice. This practice was supported by various assertions that differential discounting would lead to logical inconsistencies, including Weinstein’s and Stason’s consistency argument, Keeler’s paralyzing paradox, and Viscusi’s equivalence argument [12–14]. However, these assertions do not take account of the fact that in health economic evaluations benefits usually are measured in non-monetary terms such as quality-adjusted life years whose unit monetary value may change over time. In fact, each of these arguments can be kept up only by assuming that v_H_ remains unchanged over time [15, 16]. Another strand of the literature has provided various arguments in favor of using different discount rates (for short reviews, see [17, 18]). At the center of debate was the supposition that v_H_ would grow over time due to increasing incomes, and that this growth should be accounted for by lowering the discount rate for health [2, 5, 15, 19–27]. Meanwhile, it has been broadly accepted that the theoretical soundness of differential discounting primarily depends on whether the social objective of the healthcare decision maker is to maximize health or social welfare and whether the budget for healthcare is fixed or flexible. If the objective of the decision maker is to maximize social welfare while there is no healthcare budget constraint, a new technology should be accepted if the present v_H_ gained exceeds the present value of consumption losses, as the incremental costs of the technology do not fall on health but on consumption. Using a deterministic two-period framework, Claxton et al. [2] showed that maximizing the present v_H_ requires to discount incremental costs with the consumption discount rate, whereas the discount rate for health is equal to the consumption discount rate minus the growth rate in the v_H_ (g_V_). Our focus is on this scenario, as under these circumstances, the two-goods extension of Ramsey’s optimal growth model can be used to derive the difference between both discount rates, as shown below.

Note, however, that adjusting the discount rate for health is not imperative to reflect a changing v_H_. As a strictly equivalent alternative, one could instead inflate the future health gain according to the increase in v_H_ and then discount the adjusted health gain at the same rate as for the costs [5, 15]. The first option has become the standard approach, even if it runs the risk of confusing the relative valuation of health and consumption with time-discounting.

### Arguments in favor of an increasing consumption value of health

In the health economic literature, it is often argued that v_H_ will grow over time with increases in income [2, 15, 19], but empirical estimates of g_V_ are lacking [5]. Only a few theoretical approaches can be found aiming at giving support to the assumption of an increasing v_H_. Gravelle and Smith [15] presented two models—the first being a microeconomic model of consumer behavior and the second a welfare economic model—to argue that v_H_ grows over time. However, in both cases, the optimality conditions do not allow to strictly exclude that g_V_ is non-positive.

Hall and Jones [28] developed an economic model to explain the rising share of income devoted to healthcare by Americans during the last two decades. Essentially, they argue that the observable shift in the composition of total spending toward health expenditure is the result of an increasing v_H_. However, recent research provides increasing evidence that the income elasticity of the demand for healthcare is less than one [29–32], which is inconsistent with a basic assumption of Hall’s and Jones’ model. Moreover, their model implies that the willingness to pay (WTP) for health should rise faster than income. This is contrary to comprehensive empirical evidence, as will be shown below.

Based on a suggestion by Van Hout [25], Klok et al. [23] offered another theoretical underpinning of differential discounting. They argued that discount rates for both costs, r_C_, and health, r_H_, should be conceived as the composite of pure time preference and diminishing marginal utility of income and health, respectively. Based on this argument, they formulate r_C_, r_H_ and g_V_ as follows:

1 $$ {\text{r}}_{\text{C}} =  \uprho \, + \, \upepsilon_{\text{C}} \cdot {\text{ g}}_{\text{C}} $$

2 $$ {\text{r}}_{\text{H}} =  \uprho \, + \, \upepsilon_{\text{H}} \cdot {\text{ g}}_{\text{H}} $$

3 $$ {\text{g}}_{\text{V}} = {\text{ r}}_{\text{C}} {-}{\text{ r}}_{\text{H}} =  \upepsilon_{\text{C}} \cdot {\text{ g}}_{\text{C}} {-} \, \upepsilon_{\text{H}} \cdot {\text{ g}}_{\text{H}} , $$

where ρ denotes the rate of pure time preference, g_C_ (g_H_) the growth rate of consumption (health) and ε_C_ (ε_H_) the elasticity of marginal utility of income (health) with respect to income (health). As growth in health is probably rather small compared to income growth, the authors conclude that Eq. 2 can be expected to potentially grossly reduce to r_H_ = ρ and therefore g_V_ > 0 as far as g_C_ > 0.

This underpinning of differential discounting provides an appealing way to determine empirical estimates of r_H_ and g_V_. Moreover, it implies a strong a priori in favor of differential discounting from a purely mathematical point of view, since r_C_ = r_H_ only if, coincidentally, ε_C_ · g_C_ = ε_H_ · g_H_ [18]. However, it is not fully clear how Eqs. 1 and 2 were derived. Interpreting them as the results of a two-goods extension of Ramsey’s optimal growth model [33], as described in the next section, the first-order conditions for optimal growth are given by [34]:

4 $$ {\text{r}}_{\text{C}} =  \uprho \, + \, \upepsilon_{\text{C}} \cdot {\text{ g}}_{\text{C}} + \, \upepsilon_{\text{CH}} \cdot {\text{ g}}_{\text{H}} $$

5 $$ {\text{r}}_{\text{H}} =  \uprho \, + \, \upepsilon_{\text{H}} \cdot {\text{ g}}_{\text{H}} +_{{}} \upepsilon_{\text{HC}} \cdot {\text{ g}}_{\text{C}} $$

6 $$ {\text{r}}_{\text{C}} {-}{\text{ r}}_{\text{H}} = {\text{ g}}_{\text{V}} =  \left( {\upepsilon_{\text{C}} - \upepsilon_{\text{HC}} } \right) \, \cdot {\text{ g}}_{\text{C}} + \, \left( {\upepsilon_{\text{CH}} - \upepsilon_{\text{H}} } \right) \, \cdot {\text{ g}}_{\text{H}} $$

where ε_CH_ denotes the elasticity of marginal utility of health with respect to consumption and ε_HC_ the elasticity of marginal utility of consumption with respect to health. From this, it follows that Eqs. 1 and 2 hold only if the cross elasticities are zero, i.e. if the utility function is additively separable in health and consumption. This is a strong and counter-intuitive proposition which would need some justification in order to provide a plausible a priori.

Altogether, the health economic literature offers neither empirical evidence nor a strong theoretical a priori in support of the assumption that v_H_ will rise over time. This holds even if one is prepared to consider g_C_ > g_H_ to be a valid observation. Given this lack of evidence, we offer an additional approach to check the appropriateness of differential discounting which is based on a two-goods extension of Ramsey’s optimal growth model.

## Methods

### A two-goods extension of Ramsey’s optimal growth model

Economic theory of project evaluation postulates to discount benefits and costs to the present using the consumption discount rate [35–40]. This rate is usually approached along the lines of Ramsey’s theory of saving [33]. In this theory, the author answers the question of how much of its income a society should save to ensure optimal growth, i.e. the highest possible sum of discounted utilities of current and future generations. Ignoring uncertainty, this approach leads to the Ramsey discounting formula [33, 41]:

7 $$ {\text{r}}_{\text{t}} =  \uprho \, + \, \upepsilon \, \cdot {\text{ g}}_{\text{t}} $$

According to this formula, the discount rate applied to net benefits at time t equals the sum of the rate of the utility rate of discount and the growth rate of consumption between t and the present, weighted by (minus) the elasticity of the marginal utility of consumption.

In the original Ramsey model, all goods producing utility are aggregated into a single commodity, called consumption. This simplification does not allow accounting for changing relative prices or values of goods. In environmental economics, it is typically assumed that the growth rate of environmental goods (“ecosystem services”) will be lower than the growth rate of man-made goods. Therefore, the relative price of ecosystem services is expected to rise over time. To adequately account for the diverging growth rates across both sectors of the economy, a two-goods extension of Ramsey’s growth model has been developed. Based on this model, it has been shown that two discount rates should be applied in project evaluation, one for manufactured consumption goods, the economic discount rate, and another, the ecological discount rate, for ecosystem services [27, 34, 42–47].

The standard model to analyze the impact of a rising WTP for ecosystem services has been developed by Hoel and Sterner [44]. Their model of the economy consists of two sectors with different growth rates of consumption of manufactured goods (g_C_) and of ecosystem services (g_E_). Discounting is derived from a model of utilitarian intertemporal welfare maximization incorporating a welfare function allowing for different elasticities of utility and a variable elasticity between the consumption good C and the environmental good E. Without imposing further restrictions on the utility function, the optimal growth path implies that the economic and the ecological discount rates are given by the Eqs. 4 and 5 (substituting E for H). Assuming instead a constant elasticity between C and E simplifies Eq. 6 to

8 $$ {\text{r}}_{\text{C}} {-}{\text{ r}}_{\text{E}} = {\text{ g}}_{\text{V}} = { 1}/\sigma \, \left( {{\text{g}}_{\text{C}} {-}{\text{ g}}_{\text{E}} } \right), $$

with σ being the elasticity of substitution between C and E. Hence, a uniform social discount rate for two goods is inappropriate if both goods are less than perfect substitutes and have diverging growth rates. Instead, dual discounting should be adopted, whereat the difference between both good-specific discount rates is given by g_V_. As long as g_C_ > g_E_, r_E_ should be smaller than r_C_, with a difference between both rates increasing with decreasing σ.

Based on this model and employing the finding that for a constant elasticity of substitution (CES) utility function, the income elasticity of the WTP for the environmental good is given by the inverse of σ [48], Baumgärtner et al. [47] have determined an empirical estimate of g_V_. For Germany, they found that ecosystem services should be discounted at a rate 0.7 percentage points lower than the one for manufactured consumption goods.

### An empirical example: the case of Germany

In the following, we apply the dual discounting approach described above to empirically determine the growth rate of the v_H_ in Germany. As it is reasonable to suggest that there is some degree of substitutability between health and consumption, we assume a CES utility function to circumvent the difficulties to determine Eq. 6 empirically. Exploiting the results of Hoel and Sterner [44], g_V_ can be written as

9 $$ {\text{g}}_{\text{V}} = {\text{ r}}_{\text{C}} {-}{\text{ r}}_{\text{H}} = { 1}/\sigma \left( {{\text{g}}_{\text{C}} {-}{\text{ g}}_{\text{H}} } \right), $$

with σ being the elasticity of substitution between health and consumption. It follows that differential discounting is justified if health and consumption are less than perfect substitutes and g_C_ > g_H_. Following Baumgärtner et al. [47], we determine 1/σ empirically by imputing a value of the WTP for health.

The basic institutional features of the German statutory health insurance make Germany a good example to empirically illustrate the application of our approach. This insurance system, which is covering almost 90% of the resident population, is operated without exogenous a priori budget constraints or a fixed contribution rate imposed by some higher political authority such as the government or the parliament. Basically, the contribution rates (and hence the revenues) must be fixed at a level allowing the funds to pay for all goods and services to which the insureds are legally entitled. These entitlements include the accessibility to new healthcare technologies with proven additional benefits, but only as far as the new technologies can be provided at reasonable cost. This means that the statutory health insurance system is obliged to fund a new technology providing a health gain only if the incremental costs (which are to be financed by raising the revenues) do not represent an unreasonable burden on the community of the insureds. This decision-making perspective finds its expression most clearly in the legal provisions for the inclusion of new drugs in the benefits catalogue and the guidelines for the evaluation of new drugs. These guidelines require the conduct of a budget impact analysis as an obligatory component of the economic evaluation [49]. The combination of benefit assessment and budget impact analysis reflects the legislator’s intention to commit the decision makers in the insurance system to consider the health gains as well as the insureds’ financial capacities for consuming other goods than health.

## Results

### Findings on the growth rates of health and consumption

At present, a database to estimate a valid value of g_H_, integrating not only the changing length of life but also the change in health-related quality of life, is not available. However, given the improvements in the quantity and quality of health during the last century and the promises of modern life sciences to render the prosecution of this development possible, it is reasonable to assume that g_H_ is strictly positive. Regarding length of life, forecasts of life expectancy at birth in Germany show an increase of about one month per annual birth cohort [50]. This corresponds to an annual growth rate of length of life of roughly 0.1%; we use this value as an estimate of g_H_. Attributing a numerical value to the future growth rate of consumption (g_c_) in Germany by looking at the past would suggest a growth rate of 1.6% [14]. Alternatively, using a number derived from long-term economic forecasts [51] would amount to a somewhat lower growth rate of 1.5%. Taking the mean of these two figures as an estimate of g_C_ and the growth rate of length of life as an estimate of g_H_ (and thus neglecting the impact of changing health-related quality of life) would result in g_V_ = (1.55–0.1%) 1/σ, i.e. the growth rate of the value of health would be given as 1.45% times the value of the income elasticity of the WTP for health.

### Findings on the willingness to pay for health

To derive an estimate of the income elasticity of the WTP for health, we have exploited two strands of the empirical literature on the monetary value of health. First, we have reviewed research on the WTP for mortality and morbidity risk reductions, and second, research on the monetary value of a quality-adjusted life-year (QALY).

### The willingness to pay for mortality and morbidity risk reductions

The value of mortality risk reductions, often called the value of a statistical life (VSL) represents the rate at which an individual would trade consumption of other goods and services for small changes in their own mortality risk, given their preferences and budget constraints [52, 53]. Recent syntheses of this research containing more than 200 studies can be found in [52] and [53].

There are also numerous studies providing estimates of the income elasticity of the VSL. Instead of synthesizing the results of all individual studies, we reviewed only income elasticity estimates from meta-regressions. An important advantage of meta-analyses is that they minimize random sampling (or estimation) error by averaging across many VSL estimates [54]. Moreover, we preferred to rely on meta-analyses, as we could not find a single study reporting an estimate of the income elasticity of VSL for Germany; findings based on a broad range of different settings might be more easily transferable to Germany than results provided by single studies with their particular geographic or population characteristics. Our literature search aimed at identifying all meta-analyses of VSL published in peer-reviewed scientific journals from which it was possible to derive or calculate the income elasticity of VSL. For earlier studies, we used the results of a literature search conducted by Doucouliagos et al. [54]. This search identified 14 meta-analyses, among them 10 were published in scientific journals [55–64]. Updating the literature search in late 2017, we found six additional VSL meta-studies [54, 65–69]. The results of a total of 16 meta-regression studies are presented in a chronological order in Table 1.

**Table 1:** Income elasticities of the WTP for a reduction in mortality risks—results of meta-analyses

Author and year	Type of included studies and data base [(a): number of VSL estimates; (b): number of studies; (c): number of countries]	Results
Liu et al. 1997 [55]	WR; (a) 17;(b) 17; (c) 3	0.53, ns
Miller 2000 [56]	WR, PR, SP; (a) 68; (b) 27; (c) 13	0.85–1.00
Bowland et al. 2001 [57]	WR; (a) 33; (b) na; (c) na	1.52–2.27
Mrozek et al. 2002 [58]	WR; (a) 203; (b) 33; (c) na	0.37–0.49
De Blaeij et al. 2003 [59]	WR, SP, CPLS; (a) 95; (b) 30; (c) 10	0.50
Viscusi et al. 2003 [60]	WR; (a) 49; (b) 49; (c) 11	0.46–0.60
Kluve et al. 2008 [61]	WR, PR, SP; (a) 42; (b) 37; (c) 10	− 0.26, ns
Bellavance et al. 2009 [62]	WR; (a) 32; (b) 32; (c) 8	0.84–1.08
Lindhjelm et al. 2011 [63]	SP; (a) 856; (b) 76; (c) 24	1.17–1.31
	Reduced data set^a^: (a) 405; (b) na; (c) na	0.77–0.88
	Exclusion of VSL estimates according to authors’ recommendations: (a) 350; (b) na; (c) na	0.74–0.89
	Passed internal or external scope test: (a) 297; (b) na; (c) na	0.69–0.74
	Passed internal and external scope test: (a) 79; (b) na; (c) na	0.25–0.30
	Studies with identical questionnaire: (a) 169; (b) na; (c) na	0.30–0.44
Doucouliagos et al. 2012 [64]^b^	WR; (a) 32; (b) 32; (c) 8; re-analysis of the dataset used in [60]	0.20–0.38
Doucouliagos et al. 2014 [54]^b^	WR; (a) 101; (b) 14 meta-analyses; (c) na	0.25–0.63
Viscusi 2015 [65]^b^	WR; (a) 550; (b) 17; (c) 1	0.53–0.59
Viscusi et al. 2017 [66]^b^	WR; (a) 953); (b) 68; (c) 14	0.78
Majumder et al. 2017 [67]	WR; (a) 34; (b) 30; (c) na	0.26
Viscusi 2018 [68]^b^	WR; (a) 1025; (b) 68; (c) 14	0.53–0.85
Masterman et al. 2018 [69]^b^	SP; (a) 1145; (b) 85; (c) 27	0.69

*WTP* willingness to pay, *VSL* value of a statistical life, *WR* wage risk, *PR* product risk, *SP* stated-preference, *CPLS* cost per life saved, *ns* not significant, *na* not available

^a^VSL estimates were excluded if (a) no value for the risk change has been reported, (b) a subsample was smaller than 100 observations or the main survey sample had less than 200 observations, (c) the sample was not representative of a broad population

^b^Covariates of meta-regression include a measure of precision of the VSL estimate (usually its standard error) to control for publication selection or reporting bias

As shown in Table 1, the studies report a broad range of income elasticities, varying between − 0.26 [61] and + 2.27 [57]. However, only 3 out of the 16 analyses [57, 62, 63] report income elasticities > 1. Bowland and Beghin [57] conducted a meta-analysis of 33 wage-risk studies in industrialized countries to derive a VSL prediction function for developing economies, for which VSL estimates usually are missing. They report estimates of the income elasticity varying between 1.52 and 2.27, and thus the highest values we found in all 16 meta-analyses. This finding probably reflects that the authors were primarily interested in good predictions of low-income observations and therefore selected their preferred estimated models according to the mean-square error of prediction measures for the bottom 5 and 10 observations of their dataset. However, income elasticities of the WTP for health tend to decrease with increasing incomes [66, 69, 70]. Therefore, elasticities based on the bottom observations cannot be expected to provide sound estimates for high-income countries.

The second study reporting income elasticities > 1 was published by Lindjhem et al. [63]. Their database included 856 VSL estimates drawn from 76 stated-preference studies issued from 24 countries. For the full dataset, the authors obtained income elasticities ranging between 1.17 and 1.31. In addition, the authors tested a range of quality screening criteria to investigate the effect of limiting the meta-analysis to high-quality studies. Excluding all VSL estimates, if no value for the risk change had been reported, the estimates were based on survey samples smaller than 200 observations or subsamples smaller than 100 observations, and the sample was not representative of the broader population of the geographical area in question, resulted in income elasticities of 0.77–0.88. Similar estimates were derived when VSL estimates were excluded following authors’ recommendations. Limiting the database additionally to studies passing an internal or external scope test yielded income elasticities of 0.69–0.75. Including only studies passing both scope tests reduced the income elasticity estimates to a range between 0.25 and 0.34. Finally, limiting the data set to studies using questionnaires similar to the “good practice” questionnaire developed by Krupnick et al. [71] resulted in income elasticities of 0.30–0.44.

The third study reporting income elasticities > 1 is a meta-analysis conducted by Bellavance et al. [62]. They found elasticities between 0.84 and 1.08, depending on the number of explanatory variables included. Most interestingly, Doucouliagos et al. [64] conducted a re-analysis of their dataset to investigate the possible impact of the selection bias inherent in choosing which results to report and publish on the findings of empirical VSL research. They showed that correction for selection bias (which usually is done by augmenting the set of covariates in the meta-regression equation with the standard error of the VSL or its reciprocal) reduced the average estimate of VSL reported in [62] by 70–80%. Moreover, they found that correction for selection bias reduced the income elasticity value reported in [62] by more than half to estimates between 0.20 and 0.38. The finding that controlling for selection bias is indispensable was confirmed by Doucouliagos et al. [54], who used a data set consisting of 101 estimates of the income elasticity of VSL from 14 previously reported meta-analyses based on wage-risk studies as well as on stated-preference surveys. The authors found that correcting the selection bias transformed an average income elasticity of 0.90 to an elasticity being clearly and robustly inelastic, with a value of approximately 0.25–0.65, depending on the model specification. We could identify four additional meta-analyses controlling for publication selection and outcome reporting bias [65, 66, 68, 69]. Choosing different sets of explanatory variables, different functional forms of the meta-regression equation, and applying different econometric methods, all four studies found, without exception, income elasticities clearly smaller than 1.

Studies addressing the value of morbidity risk reductions are far less numerous and provide only very sparse and scattered evidence. A literature review conducted by Ludwig and Neumann [72] identified a total of 18 studies reporting income elasticities of the WTP for morbidity risk reductions. Eleven studies analyzed minor health problems and reported income elasticities ranging from 0.11 to 0.50. The 8 studies dealing with severe health problems found elasticities between 0.28 and 1.25, with only one estimate significantly larger than 1. Moreover, there are two meta-analyses of morbidity risk valuation studies pooling the studies included by describing the health effects of the diseases in terms of Quality of Well-Being (QWB) scores. The first study, comprising 16 contingent valuation studies on short-term air-pollution-related morbidity risks, provides income elasticities between 0.14 and 0.35, depending on the estimation method applied [73]. The second study covers a more diverse spectrum of health problems with a larger range of duration and severity [74]; based on over 230 WTP estimates from 17 different studies, an income elasticity of 0.70 to 0.88 was estimated, depending on the version of the QWB score used.

Altogether, the empirical evidence is in support of the presumption that the income elasticity of the WTP for mortality and morbidity risk reductions probably does not exceed 1. Hence, specifying the welfare function as a CES utility function implies that g_V_ is rather smaller than the difference between the g_C_ and g_H_, i.e. smaller than 1.45%, as for this function type g_V_ is given by Eq. 9.

However, some caution toward this conclusion is advisable. First, the morbidity valuation literature is rather small. In particular, there is a lack of studies regarding severe chronic diseases. Second, there are large international differences in healthcare systems, incomes, age structure, risk level and many other factors influencing the WTP for health risk reductions. Therefore, it cannot be taken for certain that the findings based predominantly on data from the US and other foreign countries (there is only a single German wage-risk study [75] included in some of the meta-analyses reviewed in this paper), can simply be transferred to Germany. Indeed, some studies demonstrate significant cross-national differences even within the group of high-income countries [76, 77]. Third, the reported WTP values usually refer to the value of one’s own health. As studies on “altruistic” WTP might well provide different results, we reviewed studies analyzing parents’ WTP for their children’s health. These studies generally show that, on average, parents value their children’s health 50–100% higher than their own health [78–85]. However, income elasticities of parents’ WTP for their children’s health reported in these studies were not larger than 0.30. These findings also are in favor of the assumption that g_V_ is positive, but smaller than the difference between g_C_ and g_H_.

### The willingness to pay for a quality-adjusted life-year (QALY)

The growing research on the monetary value of a QALY is mainly motivated by the search for a threshold value against which the results from economic evaluations expressed as costs per QALY should be judged to make decisions on the funding or reimbursement of these technologies. Such threshold values may be based on two different theoretical perspectives: the opportunity cost and the WTP approach. The WTP approach is more in line with the institutional framework of the German statutory health insurance system, in which the incremental costs associated with including the new technology into the benefit basket of the insurance system necessarily fall on consumption instead of health. In this scenario, a decision on funding a new technology should be based on comparing the cost per QALY value with the society’s WTP for a QALY (WTP-Q).

In our search for income elasticity estimates of the WTP-Q, we relied on a review of the empirical literature on the WTP-Q conducted in 2013 by Ryen and Svensson [86]. The authors identified 24 studies containing 383 unique estimates of the WTP-Q, 21 studies being direct stated-preference studies and the remaining three being VSL conversion studies. Updating the literature review in late 2017, we found only one additional study conducted by Ahlert et al. [87], the only to our knowledge that estimates WTP-Q for Germany.

To date, in contrast to the research on VSL, research on WTP-Q has not paid much attention to this value’s income elasticity. Among a total of 25 studies, only 10 reported an income elasticity of WTP-Q or enabled calculation of its value [87–96]. The main results of these 10 studies are shown in chronological order in Table 2.

**Table 2:** Income elasticities of the WTP for a QALY. Source: Own compilation or calculation

Author	Data base	Results
Studies included in Ryen and Svensson [86]		
Byrne et al. 2005 [88]	GP (130 ≤ n ≥ 160), USA	0.16–0.27
King et al. 2005 [89]	Hospital patients (n = 391), USA	0.30–0.87
Bobinac et al. 2010 [90]	GP (n = 1091), The Netherlands	0.05–0.21^a^
Shiroiwa et al. 2010 [91]	GP (504 ≤ n ≥ 1114), Japan, South Korea, Taiwan, Australia, United Kingdom, USA	J: 0.37 SK: 0.53 T: 0.69 AUS: 0.73 UK: 0.77 USA: 1.02
Haninger et al. 2011 [92]	GP (n = 2858), USA	0.02–0.15
Zhao et al. 2011 [93]	GP (n = 364), China	0.00–0.02^a^
Bobinac et al. 2013 [94]	GP (n = 1004), The Netherlands	0.17–0.23^a^
Thavorncharoensap et al. 2013 [95]	GP (n = 1191), Thailand	0.31
Shiroiwa et al. 2013 [96]	GP (n = 2283), Japan	0.03–0.16
Studies not included in Ryen and Svenson [86]		
Ahlert et al. 2016 [87]	GP (507 ≤ n ≥ 1501), Germany	0.12–0.40

*GP* general population (adults), *n* number of study participants, *QALY* quality-adjusted life year, *WTP* willingness to pay

^a^Arc elasticities, calculation based on the means of the two middle-income categories

Table 2 shows that the estimates of the income elasticity of the WTP-Q found in the 10 studies range between 0.00 and 1.02; most estimates are substantially smaller than 1. The German study reports income elasticities ranging between 0.12 and 0.40, depending on special features of the study design [87]. As is the case with VSL studies, almost all studies deal with one’s WTP for one’s own health. However, the WTP for a health gain achieved anywhere in society might provide the more relevant information for societal decision-making in collectively funded healthcare systems. Bobinac et al. have estimated both the “individual value” [90] as well as the “social value” of health [94]; apparently, they found no relevant differences in the income elasticities of the two WTP-Q values.

### A plausible range of the growth rate of the WTP for health

Assembling the data from studies on the WTP for mortality and morbidity risk reductions and studies on the WTP-Q suggests health to be a normal good, as almost all studies reported positive income elasticities. Concurrently, the vast majority of studies reported income elasticities considerably smaller than 1, indicating health to be a necessity rather than a luxury. Combining this finding with the values for g_C_ and g_H_ suggests, for Germany, an upper bound of g_V_ of about 1.5% per year. On the other hand, as studies reporting income elasticities lower than 0.2 are relatively rare, supposing a lower bound in the magnitude of 0.3% per year appears reasonable.

## Discussion

There is a wide consensus that if the objective of the healthcare decision maker is to maximize social welfare, costs and health effects should be discounted at different rates if the growth rates of health and consumption differ. Typically, it is argued that since the health of the population is expected to grow more slowly than its consumption of goods and services, as time passes, the welfare gain from better health will increase relative to the welfare from increases in consumption, and therefore the value of health in terms of consumption will increase [2]. However, a more thorough look at the various arguments in the health economic literature aiming at giving support to the assumption of an increasing v_H_ reveals that there is no strong theoretical a priori and no empirical evidence at all in favor of this assumption. Therefore, we added a further approach to account for different growth rates of health and consumption when setting the discount rates. Based on a two-goods extension of Ramsey’s optimal growth model and the assumption that the social planner’s preferences might be depicted by a CES utility function, g_V_ is given by the product of the income elasticity of the WTP for health and the difference between the growth rate of consumption and the growth rate of health [44].

To illustrate the application of our approach with an empirical example, we chose Germany as a country meeting the assumptions on which the results of the analysis by Claxton et al. [2] are based. By doing this, we made the plausible assumption that the minimum value of g_H_ is given by the growth in life expectancy at birth. As an estimate of the future g_C_, we took the mean of a retrospective analysis and a long-term forecast. Reviewing the literature on the WTP for mortality and morbidity reductions as well as for QALYs, we found that the vast majority of studies reported income elasticities larger than 0.2 and smaller than 1. This suggests a range of g_V_ between 0.3 and 1.5%.

Our approach has the advantage that it does not make use of ad hoc arguments in favor of differential discounting, but is embedded in Ramsey’s optimal growth model, which to date, is regarded by the majority of economists as the appropriate theoretical framework for deriving the discount rate to be applied in the economic evaluation of public projects [41]. There are, however, two major limitations associated with the theoretical underpinning of our approach to gain an empirical estimate of g_V_. First, the choice of a CES utility function which has been made to facilitate the difficulties to gain an empirical estimate of g_V_ cannot be justified by providing strong arguments that the social planner’s preferences are or should be depicted by this type of function; in fact, the wide use of CES functions in economic theory owes simply to their tractability. However, the limitation introduced by assuming a constant instead of a varying elasticity between health and consumption should not be overemphasized, as economic evaluation of healthcare technologies typically deals with marginal projects. Second, the two-goods extension of Ramsey’s optimal growth model which we have applied to estimate g_V_ ignores all aspects of uncertainty. Perhaps the most important source of uncertainty over a long time horizon is the growth rate of consumption, g_C_. Within the theoretical framework developed by Hoel and Sterner it is, however, not obvious whether and how uncertainty about g_C_ will affect g_V_ [44].

In addition, there are several data limitations suggesting that our estimates of the range of g_V_ should be considered only as a starting point for further research. First, the range of a 0.3 to 1.5% annual growth rate of the consumption value of health (g_v_) does not take into account the growth of health that is due to the probable improvement of health-related quality of life, which could shift the range toward lower values. Therefore, future research should call more attention to explore feasible options on how to integrate health-related quality of life into measures of population health. Second, the overwhelming majority of studies estimating income elasticities of the WTP for health aimed at determining the WTP for one’s own health. However, even accepting the view that political decisions should reflect individual preferences, one may take the view that it is the WTP not only for one’s own but for others’ health as well that should count for informing these decisions. Therefore, more research on the WTP for improving population health is needed. Finally, the total of studies on which our estimate of the range of g_V_ is based includes only one study on the income elasticity of the WTP for health in Germany. Therefore, our estimate rests on the untested assumption that foreign findings can be transferred to Germany. Given these limitations as well as some other unsolved problems, such as defining quality criteria for selecting an appropriate study sample, we refrained from doing an own meta-analysis in order to further close the plausible range of g_V_.

Notwithstanding these limitations, we are confident that the currently available empirical evidence makes the case for a growing consumption value of health. Given this finding, the current practice of applying the same discount rate to costs and health gains introduces a systematic bias against healthcare technologies with upfront costs and long-term health effects. Thus, in principle, differential discounting with a lower rate for health effects appears to be a more appropriate discounting model for base-case analyses in health economic evaluations. However, to gain a more precise value of g_V_, more research on the consumption value attributed to health in Germany is necessary.

## Conclusion

Applying the two-goods extension of Ramsey’s optimal growth model, we have generated a range for an empirical estimate of the growth rate of the consumption value of health, g_v_, in Germany. The currently available empirical evidence suggests that the income elasticity of the WTP for health is probably not larger but rather smaller than 1 and probably not smaller but rather larger than 0.2. Based on this finding, the growth rate of the consumption value of health might reasonably be assumed to be within a range of 0.3 to 1.5%. This implies that in health economic evaluations, the discount rate applied to health gains should be 0.3 to 1.5 percentage points lower than the discount rate applied to costs. Of course, these figures provide only a starting point for further research on the value of health and its change over time, not least because up to now data on the income elasticity of the WTP for health in Germany are sparse.

## Data Availability

Not applicable.

## Abbreviations

CES: constant elasticity of substitution
CPLS: cost per life saved
GP: general population
n.a.: not available
n.s.: not significant
PR: product risk
QALY: quality-adjusted life-year
QWB: quality of well-being
SP: stated preference
VSL: value of a statistical life
WR: wage risk
WTP: willingness to pay
WTP-Q: willingness to pay for a quality-adjusted life-year
